# Intracellular Transport in Cancer Metabolic Reprogramming

**DOI:** 10.3389/fcell.2020.597608

**Published:** 2020-10-30

**Authors:** Marte Sneeggen, Noemi Antonella Guadagno, Cinzia Progida

**Affiliations:** Department of Biosciences, University of Oslo, Oslo, Norway

**Keywords:** membrane trafficking, cancer cell metabolism, cell proliferation, epithelial to mesenchymal transition, invasion

## Abstract

Tumor progression is a complex process consisting of several steps characterized by alterations in cellular behavior and morphology. These steps include uncontrolled cell division and proliferation, invasiveness and metastatic ability. Throughout these phases, cancer cells encounter a changing environment and a variety of metabolic stress. To meet their needs for energy while they proliferate and survive in their new environment, tumor cells need to continuously fine-tune their metabolism. The connection between intracellular transport and metabolic reprogramming during cancer progression is emerging as a central process of cellular adaptation to these changes. The trafficking of proteolytic enzymes, surface receptors, but also the regulation of downstream pathways, are all central to cancer progression. In this review, we summarize different hallmarks of cancer with a special focus on the role of intracellular trafficking in cell proliferation, epithelial to mesenchymal transition as well as invasion. We will further emphasize how intracellular trafficking contributes to the regulation of energy consumption and metabolism during these steps of cancer progression.

## Introduction

During cancer progression, tumor cells go through different stages, which are defined as hallmarks of cancer. One of the main hallmarks is the ability to sustain proliferation. Misregulation of growth-promoting signals stimulates cell survival and energy metabolism, resulting in tumor growth (Hanahan and Weinberg Robert, 2011). As cancer further develops, cells may become able to disseminate from the primary site of origin. This is usually induced by loss of epithelial markers such E-cadherin, and characterized by the transition from an epithelial phenotype to a mesenchymal phenotype, a process known as epithelial to mesenchymal transition (EMT). Genes that in normal tissues express molecules involved in cell-to-cell adhesion and cell-to-extracellular matrix adhesions are altered in highly aggressive carcinomas, typically downregulated (Hanahan and Weinberg Robert, 2011). After losing cell-cell adhesions, cancer cells acquire migratory ability, leading eventually to invasion into neighboring tissues and forming metastatic sites in distant organs (Son and Moon, 2010).

During these transitions, cancer cells undergo metabolic changes, which allow them to satisfy their increased need of energy. The reprogramming of energy metabolism is now recognized as one of the hallmarks of cancer. One of the most known metabolic adaptation in malignant cells is the Warburg effect, that is characterized by increased glucose uptake and lactate production in the presence of oxygen (Potter et al., 2016). To satisfy their high nutritional and energetic requirements, cancer cells exploit intracellular trafficking pathways such as macropinocytosis and autophagy to scavenge the tumor microenvironment for nutrients and macromolecules. This fuels the cells to sustain proliferation, undergo EMT, as well as drive invasion. In this review, we will highlight the contribution of the intracellular transport for the metabolic adaptations required during the different stages of tumor progression.

## Intracellular Trafficking in Cancer Cell Proliferation

One of the first challenges that cancer cells overcome during cancer transformation is the ability to sustain chronic proliferation (Hanahan and Weinberg Robert, 2011). Keeping up with a sustained proliferation signal has a cost in term of energy requirement. To be able to proliferate, cells must duplicate their mass. Therefore, they need to reprogram their metabolism to meet the need for larger amount of nutrients to support the synthesis of new macromolecules (Davidson and Vander Heiden, 2017).

**FIGURE 1:**
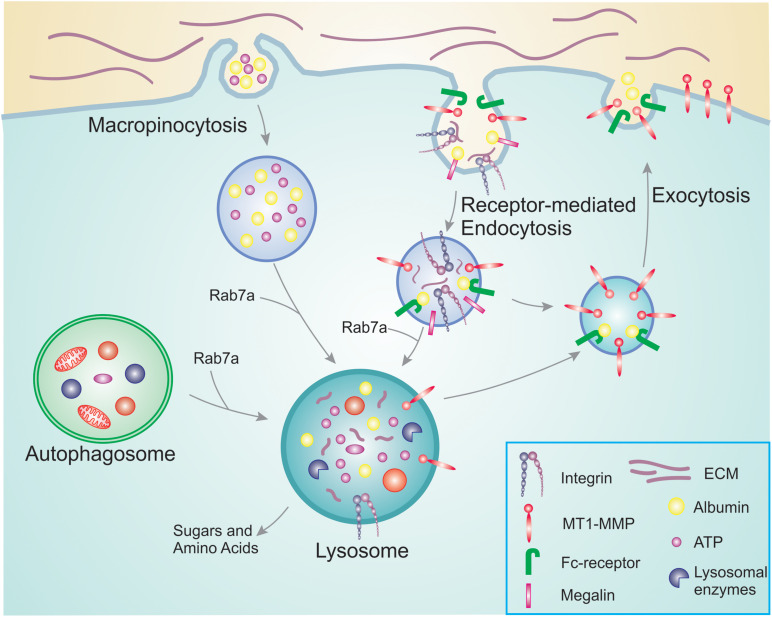
Overview of nutrient scavenging pathways in cancer cells. During cancer progression, cancer cells increase their energy and nutrient requirement to meet the demand of constant proliferation and sustain processes such as migration and invasion. For this, they use membrane trafficking pathways to scavenge nutrients already available. Macropinocytosis allows the bulk internalization of extracellular ATP as well as albumin and other nutrients. In a similar manner, receptor-mediated endocytosis is responsible for the uptake of receptors and their ligands that will be degraded in the lysosomes providing new building blocks to be reused. Examples are integrins that bind to ECM components, and megalin which binds to albumin in the extracellular environment. The internalized albumin can either bind to neonatal Fc receptor inside the endosomes and be recycled back to the plasma membrane, or degraded in the lysosomes. To access the pool of nutrients already available in the cell, cancer cells can hijack autophagy. Engulfed damaged organelles and protein aggregates can thus be broken down and degraded in the lysosomes for reuse. The altered metabolism during cancer progression results in increased MT1-MMP recycling, thus promoting cell invasion.

Cells that are proliferating rapidly have different metabolic needs than those that are in a resting state. Even though glucose and glutamine have been believed to be the major source of energy, it is now clear that the cells also use nutrients and amino acids available in the environment rather than synthesizing them *de novo*. This is a more convenient strategy as *de novo* synthesis requires more energy compared to reusing already existing nutrients and amino acids (Hosios et al., 2016). Pre-existing nutrients necessitate to be transported either from the extracellular environment or from other cellular compartments inside the cells to lysosomes for their degradation into recyclable building blocks (Davidson and Vander Heiden, 2017; Figure 1).

### Macropinocytosis and Cancer Cell Proliferation

Cancer cells have an increased need for nutrients and therefore adopt different strategies to access macromolecules from the tumor microenvironment. Macropinocytosis is an effective and rapid way to internalize macromolecules from the environment. It is an actin-dependent endocytic mechanism consisting of non-specific uptake of large amounts of extracellular fluid and nutrients into large vesicles (King and Kay, 2019). This process is crucial for nutrient uptake to support tumor cell fitness and it is associated with cell growth. Indeed, pharmacological inhibition of macropinocytosis suppresses tumor growth and it has been suggested that this process could be a possible target for anticancer therapies (Commisso et al., 2013). Macropinocytosis also boosts intracellular Adenosine triphosphate (ATP) concentration by directly ingesting ATP molecules when they are available in the extracellular environment (Figure 1; Qian et al., 2014). Extracellular ATP concentration in tumors is up to 10^4^ times higher than in normal tissues (Pellegatti et al., 2008; Falzoni et al., 2013; Qian et al., 2014). This extracellular ATP increases the survival of cancer cells during metabolic stress by protecting against tumor inhibition drugs. It has been suggested that following ATP internalization, the increased intracellular ATP interferes with tumor inhibition drugs that compete with ATP for their anticancer activity (Qian et al., 2014). In line with this, extracellular ATP reduced the function of the cancer drug sunitinib that works as an ATP competitor targeting receptor tyrosine kinases (Papaetis and Syrigos, 2009; Qian et al., 2014).

After internalization, macropinosomes deliver their content to the lysosomes where the internalized macromolecules are broken down. The obtained amino acids provide a carbon source to the central metabolism, and serve as building blocks for protein synthesis during proliferation in conditions lacking free amino acids (Palm, 2019). The small GTPase Rab7a, which regulates the fusion between endosomes and autophagosomes with lysosomes, is enriched in melanoma cells where it also is important for sustaining cell proliferation and cancer progression (Alonso-Curbelo et al., 2014, 2015). In particular, at early stages of melanoma development, Rab7a is upregulated and sustains melanoma cell proliferation controlled by the lineage-specific transcription factor SOX10 and the oncogenic transcription factor MYC, which is activated at early stages of melanoma development (Alonso-Curbelo et al., 2014). Rab7a upregulation at these early stages of melanocyte transformation hyperactivates Rab7a-mediated lysosomal degradation to counteract the enhanced macropinocytic influx associated with oncogene-induced senescence (Alonso-Curbelo et al., 2015). During melanoma progression, Rab7a expression is then downregulated. In highly invasive melanoma cells, this favors invasive phenotypes supporting Rab7a as a risk factor for melanoma metastasis and poor survival (Alonso-Curbelo et al., 2014).

Nutrient scavenging consists in the uptake of macromolecules from the extracellular environment and their degradation to produce ATP or to be used in anabolism (Finicle et al., 2018). Scavenging is controlled by the mechanistic target of rapamycin complex-1 (mTORC1) and AMP kinase (AMPK), which are involved in the regulation of macropinocytosis (Swanson and King, 2019). AMPK activates all forms of scavenging, while mTORC1 represses the effect of scavenging by interfering with the catabolism happening in the lysosomes (Finicle et al., 2018). AMPK regulates diverse metabolic cellular processes, and also endocytic traffic during metabolic stress. When it is activated, it inhibits energy demanding processes and enhances catabolic reaction to generate ATP (Rahmani et al., 2019). AMPK can both suppress but also promote tumor growth (Hardie, 2015). It has been suggested that this depends on the timing of modification, mutation and overexpression of AMPK or of the upstream kinase Liver kinase B1 (LKB1). In the initial steps of cancer, inactivation of this pathway may help cell growth by utilizing anabolic pathways. In later stages, activation of the LKB1–AMPK pathway could protect the tumor cells against oxidative stress by facilitating metabolic adaptations (Jeon, 2016).

mTORC1 is a signaling hub that coordinates nutrient status and cell growth. Activated mTORC1 regulates cellular metabolism and growth by stimulating protein synthesis (Kim and Guan, 2019). The internalization of amino acids in macropinosomes and their delivery to the lysosomes is essential for mTORC1 growth factor-dependent activation (Yoshida et al., 2015). The control of mTOR signaling is critical for the cells and its dysregulation leads to several diseases such as cancer, diabetes, and metabolic diseases (Yoshida et al., 2015).

Amino acid depletion stimulates macropinocytosis and the scarcity of glutamine drives this process (Lee et al., 2019). The nutrient stress triggered from amino acid depletion enhances epidermal growth factor (EGF) receptor signaling that in turn increases macropinocytosis by regulating membrane ruffling and cytoskeleton dynamics (Lee et al., 2019). The activation of the actin cytoskeleton occurs through the small GTPase Ras (Bloomfield and Kay, 2016; Recouvreux and Commisso, 2017). Ras is frequently mutated in cancer and activated in almost 33% of all human cancer (Bloomfield and Kay, 2016; Lanfredini et al., 2019). Ras-driven cancer cells have a higher rate of macropinocytosis. Over-activation of Ras promotes metabolic rewiring and cell proliferation not only by activation of macropinocytosis to internalize extracellular nutrients and enhancing uptake of glucose, contributing to the Warburg effect, but also by inducing autophagy (Recouvreux and Commisso, 2017; Palm, 2019).

Ras-transformed cancer cells are able to take up albumin through macropinocytosis. Degradation of albumin is a source of glutamine, one of the most deprived nutrients in cancer environments. Hence, the macropinocytic uptake of albumin could serve to sustain the proliferation of oncogenic Ras cells by constituting a source of amino acid supply (Commisso et al., 2013; Ha et al., 2016; Palm, 2019). Glutamine serves indeed as important source of carbon, which in different tumors is utilized for TCA cycle anaplerosis. In proliferating cells, glutamine-dependent anaplerosis is critical for mitochondrial metabolism and essential for cell growth (Cluntun et al., 2017). The internalization of albumin through macropinocytosis and the downstream use of albumin-derived amino acids as a source of energy seems to be a unique property of cancer cells, since normal cells adjacent to a tumor lack this ability (Davidson et al., 2017). The stimulation of macropinocytosis in cancer cells is, however, not limited to Ras-transformed cells, as activating mutations in Src kinases also drive macropinocytosis (Amyere et al., 2000; Finicle et al., 2018).

Intriguingly, it has been recently demonstrated that under nutrient-limited conditions, cancer cells within pancreatic ductal adenocarcinoma are able to internalize collagen fragments through macropinocytosis. This extracellular matrix protein represents a proline reservoir that is used as a nutrient source in the absence of other fuels. In this way, the collagen-derived proline contributes to promoting cancer cell survival as well as cell proliferation (Olivares et al., 2017). Therefore, it seems that cancer cells have developed an efficient strategy to obtain nutrients from alternative sources through macropinocytosis followed by lysosomal degradation of extracellular proteins. This allows to furnish the energy and nutrient demand for sustained proliferation.

### Receptor-Mediated Internalization for Nutrient Scavenging

Nutrient scavenging does not only occur by macropinocytosis. Cancer cells can also utilize receptor-mediated scavenging to sustain their growth and proliferation (Figure 1). An example of receptor-mediated scavenging is represented by the integrin-mediated endocytosis of extracellular matrix (ECM) components (Finicle et al., 2018). Integrins are cell surface receptors for ECM components that link the actin cytoskeleton to the ECM. During cancer progression, the trafficking of integrins is often upregulated resulting in the internalization of the receptors but also of the ECM components bound to the integrins. The ECM consists of collagen, laminin, and fibronectin. These extracellular proteins are also heavily glycosylated, thus ECM scavenging yields amino acids and sugars to sustain cell proliferation. Dietary restriction and nutrient deprivation induces laminin scavenging by integrin α6β4-mediated endocytosis. Laminin degradation in the lysosomes enhances mTORC1 signaling, preventing cell death and promoting cell survival (Muranen et al., 2017). Similarly, in ovarian cancer cells, integrin α5β1 binds fibronectin, which is then internalized and degraded in the lysosomes, and the resulting amino acids activate mTORC1 (Rainero et al., 2015). However, the mechanisms for integrin-mediated nutrient scavenging in tumors are still poorly characterized and further studies are required to better understand this process.

It is not only integrins and ECM that are involved in nutrient scavenging by receptor-mediated endocytosis. Also albumin is endocytosed upon binding to megalin or other cell surface scavenger receptors. The internalized albumin can either bind to neonatal Fc receptor (FcRn) in endosomes and be recycled back to the plasma membrane, or degraded in the lysosomes. Degradation of albumin results in increased amino acid and possibly also lipid availability (Finicle et al., 2018).

### Autophagy and Cancer Cell Proliferation

Autophagy is another process that provides nutrients and energy to the cell. It is used by cells to recycle their own compartments after sequestering them in a double membrane organelle, the autophagosome. When nutrients are running low, autophagosome formation is initiated to engulf macromolecules, protein aggregates and damaged organelles from the cytosol (Davidson and Vander Heiden, 2017). The autophagosomes then fuse with the lysosomes. Degradation inside the lysosomes provides the cells with new building blocks for protein synthesis (Kimmelman and White, 2017).

Upregulation of autophagy can occur in response to hypoxia or metabolic stress to ensure survival. Mice that lack essential autophagy genes such as *Atg5* and *Atg7*, die from nutrient starvation underlying how essential autophagy is to provide nutrients during metabolic stress (Kuma et al., 2004). Similar to macropinocytosis, autophagy allows the cells to tap into a pool of macromolecules. Both pathways are exploited by cancer cells to obtain nutrients for survival and growth. The main difference is that macropinocytosis internalizes nutrients from the extracellular environment, while autophagy utilizes what is already available inside the cells (Palm, 2019). The importance of autophagy in cancer cell proliferation, therefore, seems to be connected to its role in supporting tumor metabolism. In line with this, mutations in the Ras pathway are often associated with high levels of autophagy required to maintain cancer cell metabolism (Guo et al., 2011; Lock et al., 2011; Yang et al., 2011).

Essential autophagy genes such as *beclin-1*, which is important in the formation of the autophagosome, are upregulated in several types of cancers, including colorectal and gastric cancer (Ahn et al., 2007). Furthermore, Rab escort protein 1 (REP1) is associated with cancer progression by contributing to cell growth and survival through the regulation of mTOR signaling and its downstream pathways (Choi et al., 2017). REP1 is involved in the recruitment of Rab proteins to membranes as well as in the regulation of autophagy (Alexandrov et al., 1994; Choi et al., 2017). Knockdown of REP1 suppresses mTOR activity, blocking autophagy and increasing macropinocytosis. Even though the exact mechanism used by REP1 to regulate autophagy is not known, it is suggested that REP1 modulates the localization of lysosomes and mTOR thereby affecting their activity. It is also reasonable to think that REP1 controls the recruitment of Rab proteins necessary for this process, such as Rab7a (Choi et al., 2017).

The role of autophagy in cancer is quite complex and not fully elucidated yet. It seems to be dependent on several factors such as the type of tumor or the cancer stage. In the initial stages of cancer, autophagy acts as a tumor suppressor through quality control of proteins and removing damaged organelles and protein aggregates (Mathew et al., 2009; Li et al., 2020). By controlling these events, it can prevent sustained proliferation and therefore tumor initiation. However, in the later stages, when a tumor has formed, autophagy can protect cancer cells by helping them cope with cellular stress using the same strategies as in the early phases (Li et al., 2020). Autophagy seems also to contribute to the ability of cancer cells to develop resistance to chemotherapy by protecting them from the stress inflicted by the therapy (Sui et al., 2013; Ma et al., 2014).

## Epithelial to Mesenchymal Transition

**FIGURE 2:**
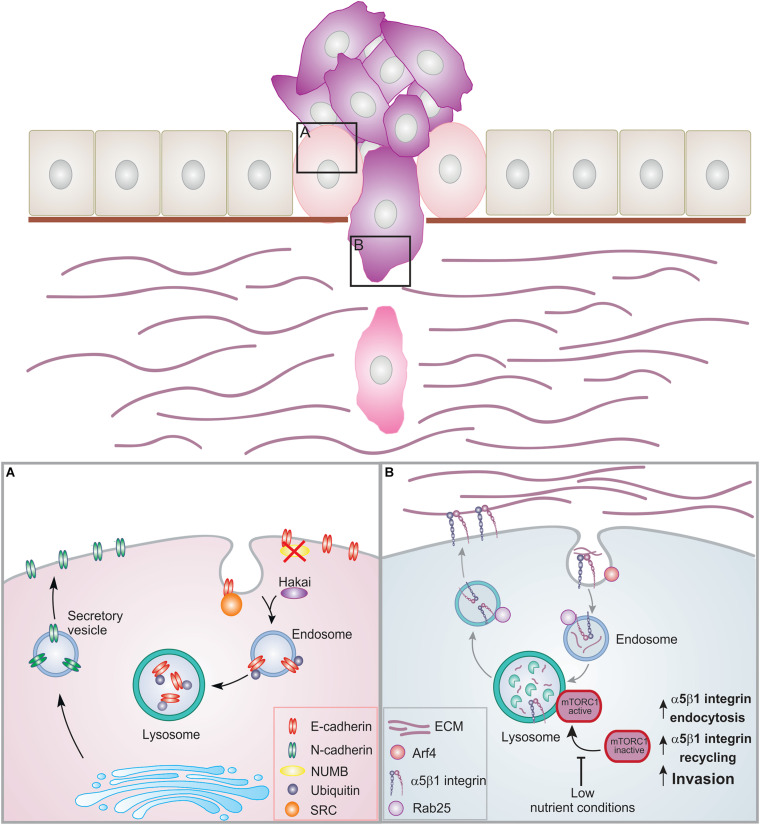
Membrane trafficking events in EMT and cell invasion. Epithelial cells are organized in layers by establishing contacts with neighboring cells as well as the basement membrane. During cancer progression, the cells can lose these contacts leading to EMT and increased proliferation. Cancer cells can then acquire migratory ability, breaching through the basement membrane and invading into the ECM. **(A)** During EMT, E-cadherin is internalized and degraded. NUMB regulates the internalization and recycling of E-cadherin. When NUMB is lost, E-cadherin relocalizes to the apical side. Upon internalization, E-cadherin can be phosphorylated by src leading to ubiquitination by the E3 ligase Hakai. Ubiquitinated E-cadherin is delivered to lysosomes for degradation. This results in the loss of adherens junctions. N-cadherin is then transported to the surface promoting migration. **(B)** Trafficking of α5β1 integrin under low nutrient conditions. Inhibition of the mTORC1 activation and of its recruitment to lysosomes promotes Arf4-dependent endocytosis, α5β1 integrin recycling, and cell invasion.

The ability of cells to change their morphology and phenotype is crucial during embryonic development but also in tissue repair in adults. Transitions from epithelial to mesenchymal cells and back again are known as cellular plasticity (Corallino et al., 2015). Several cancers derive from epithelial cells. These cells are the building blocks of most organs and are organized in tissues by establishing contacts with their neighboring cells. When epithelial cells transition to cancer cells, they lose their epithelial phenotype and acquire a mesenchymal phenotype during a process called epithelial to mesenchymal transition (EMT) (Sciacovelli and Frezza, 2017). During this process, epithelial cells lose their junctions and apical-basal polarity, re-organize their cytoskeleton, change signaling programs and alter gene expression. This results in the loss of contacts with the neighboring cells leading to increased motility of individual cells and in the development of an invasive phenotype. EMT is indeed an essential step in cancer cell progression, which leads to invasion and metastasis (Figure 2).

When cells undergo EMT, their metabolic needs become different (Kang et al., 2019). Shaul et al. (2014) found a mesenchymal metabolic signature consisting of 44 upregulated metabolic genes that are essential for EMT but not for cell proliferation. The reprogramming of gene expression has indeed a crucial role during EMT. However, also non-transcriptional changes, including alteration of intracellular trafficking, play a vital part in this process (Le Bras et al., 2012).

In epithelial cells, adherens junctions maintain cell-cell adhesion by connecting transmembrane proteins to the actin cytoskeleton. EMT is characterized by the loss of E-cadherin, one of the major structural components of these junctions. In normal cells, E-cadherin is rapidly internalized from the cell surface and then recycled back to form new cell-cell contacts (Lu et al., 2003; Palacios et al., 2005). However, the endocytic pathway is often dysregulated in cancer, with a shift in the balance between recycling and degradation. In the early phases of EMT, adherens junction dissociation often occurs as result of changes in E-cadherin transport, leading to the internalization of E-cadherin followed by its transportation and degradation into lysosomes (Janda et al., 2006; Ulrich and Heisenberg, 2009; Le Bras et al., 2012). The endocytosis and recycling of E-cadherin is regulated by its interactor NUMB. Loss of NUMB causes E-cadherin to relocate and accumulate at the apical side decreasing cell-cell adhesion, and promoting cell migration (Wang et al., 2009). In line with this, in triple-negative breast cancer, an aggressive type of cancer, reduced NUMB expression is often associated with elevated EMT (Zhang et al., 2016).

During EMT, activated v-Src, a kinase involved in oncogenesis, phosphorylates E-cadherin. After phosphorylation, the E3 ligase Hakai catalyzes the ubiquitination of E-cadherin leading to the trafficking and degradation of E-cadherin to lysosomes (Fujita et al., 2002). Even though the role of Hakai in E-cadherin ubiquitination in physiological conditions remains unclear (Niño et al., 2019), it is intriguing that the expression of this ligase is gradually increased during the different stages of colon cancer progression, which is in line with its suggested role in E-cadherin modulation in EMT (Castosa et al., 2018). Downregulation of E-cadherin facilitates a switch to N-cadherin, which is associated with enhanced migration and invasion (Figure 2A; Loh et al., 2019). Recently, it has been demonstrated that E-cadherin impacts cell metabolism as mechanical forces exerted on E-cadherin activates AMPK thereby stimulating actomyosin contractility, glucose uptake and ATP production (Bays et al., 2017).

One of the major EMT inducers is the transforming growth factor beta (TGF-β) (Katsuno et al., 2013; Corallino et al., 2015). Internalization of the TGF-β receptor triggers a series of downstream cascades, which eventually lead to exocytosis of ATP containing vesicles (Cao et al., 2019). The released ATP functions as an extracellular messenger. It binds to and activates the purinergic receptor P2X7, resulting in EMT induction by upregulating mesenchymal markers and downregulating epithelial markers (Cao et al., 2019). The extracellular ATP thus induces metalloproteinase expression, but it also serves as an energy source for cell detachment. This extracellular ATP is indeed internalized by macropinocytosis, providing the energy required to allow morphological changes and movement (Cao et al., 2019).

## Intracellular Trafficking and Energy Requirement in Cell Invasion

Cancer cells that have undergone EMT experience changes that include not only the loss of adherens junctions and apical-basal polarity, but also the re-organization of their cytoskeleton and morphology. This leads to the acquisition of migratory ability that can develop in an invasive phenotype.

For efficient cell migration, adhesion molecules such as integrins are rapidly internalized and transported along the endosomal system. To evade lysosomal degradation, integrins are recycled back to the plasma membrane. This replenishes the plasma membrane pool of integrins and promotes their rapid turnover for cell migration (Mosesson et al., 2008; Dozynkiewicz et al., 2012; Sun et al., 2018; Moreno-Layseca et al., 2019). Therefore, it is not surprising that altered integrin trafficking is linked to invasive processes (Hamidi and Ivaska, 2018).

For example, gain-of-function mutant proteins of the tumor suppressor p53, which are often associate with cancer, increase α5β1 integrin recycling (Muller et al., 2009). α5β1, together with Rab-coupling protein (RCP; also known as Rab11-FIP1), recruits receptor tyrosine kinases, regulating their recycling and potentiating downstream signaling via protein kinase B (PKB)/Akt, thus resulting in invasive migration (Caswell et al., 2008; Muller et al., 2009; Jacquemet et al., 2013). RCP-driven endocytic recycling of α5β1 integrin enhances invasive migration of cancer cells by reprogramming the actin cytoskeleton to promote the formation of cell protrusions and actin-related protein 2/3 (Arp2/3) complex-independent cancer cell invasion *in vivo* (Jacquemet et al., 2013; Paul et al., 2015). Furthermore, mutant p53 increases the expression of the motor protein myosin X, which binds to β1 integrin to mediate its transport to filopodia (Arjonen et al., 2014). It has therefore been suggested that blocking α5β1-integrin might have therapeutic benefit in mutant p53-expressing cancers (Muller et al., 2009).

Gain-of-function p53 mutants, by promoting glucose transporter 1 (GLUT1) translocation to plasma membrane, stimulate glucose uptake, glycolysis and thus, the Warburg effect (Zhang et al., 2013). Hence, mutant p53, by affecting different intracellular transport pathways, coordinates cell metabolism and integrin recycling to promote cell invasion.

Small GTPases, along with their effectors, control integrin recycling with huge impact on cell invasion. Rab11- and Arf6- dependent recycling of integrins is associated with cancer invasion (Yoon et al., 2005; Das et al., 2018; Moreno-Layseca et al., 2019). A signaling pathway involving phosphorylation of Rab34 inhibits β3 integrin lysosomal degradation mediating its recycling back to the plasma membrane to promote cell migration (Sun et al., 2018). In triple-negative breast cancer cells, Rab5a stimulates Rab4-dependent fast recycling of α5β3 integrin, thus leading to cell invasion (Frittoli et al., 2014; Linder and Scita, 2015). Furthermore, Rab25, which directly associates with integrin α5β1, promotes integrin recycling from late endosomes/lysosomes at the cell front to drive invasion (Caswell et al., 2007; Dozynkiewicz et al., 2012).

Ligand-engaged α5β1 integrin are trafficked under control of Rab25 to late endosomes/lysosomes following Arf4-dependent internalization. This pathway is necessary to maintain lysosomal positioning at the perinuclear region and to recruit and activate the nutrient sensor mTORC1 on lysosomes. Interestingly, in response to low-nutrient status of cancer cells, the recruitment of mTOR to late endosomes/lysosomes is inhibited, further promoting ligand-bound α5β1 internalization and trafficking to lysosomes. This stimulates the degradation of ECM components in the lysosomes as well as Rab25-mediated α5β1 integrin recycling at the plasma membrane (Dozynkiewicz et al., 2012; Rainero et al., 2015). Consequently, this pathway connects nutrient sensing to ECM internalization and integrin recycling to promote cell invasion (Figure 2B).

Degradation of ECM components during cancer invasion occurs not only in the lysosomes but also outside the cell. Diverse polarized trafficking pathways converge at the invadopodia, plasma membrane protrusions responsible for ECM degradation and invasion, for the local delivery of proteolytic enzymes, which have pivotal role in defining the malignant features of cancer cells. Indeed, extracellular degradation-mediated cell invasion is carried by proteolytic enzymes, such as cathepsins and matrix metalloproteinases (MMPs), which can be trafficked either through the secretory pathway or via exocytosis of peripheral lysosomes (Bonnans et al., 2014).

Rab5-mediated endocytosis regulates the internalization and delivery of the membrane-associated MT1-MMP (membrane-type 1 matrix metalloproteinase), an important invasion-promoting enzyme, to non-degrading Rab7a-positive endosomal reservoirs before being exocytosed at invadopodia for ECM degradation (Planchon et al., 2018). A further regulation of MT1-MMP recycling to the plasma membrane has been described to be dependent on WDFY2 and Rab4 following a VAMP3-dependent mechanism (Sneeggen et al., 2019). However, while recycling is considered the major route for fast delivery of proteases to the plasma membrane for ECM degradation, MT1-MMP can additionally be delivered to the plasma membrane following Rab8-dependent polarized exocytosis (Bravo-Cordero et al., 2007) or Rab27-dependent exosomal release (Hoshino et al., 2013). High levels of glutamine consumption contribute to cancer aggressiveness by generating a source of extracellular glutamate. This extracellular glutamate activates its receptor GRM3 on the plasma membrane, stimulating Rab27-mediated recycling of MT1-MMP to promote invasiveness (Dornier et al., 2017). This highlights how changes of tumor environment such as the increased extracellular glutamate and low-nutrient status of cancer cells alter cellular metabolism leading to aberrant endosomal recycling to drive cell invasion.

In addition to have a role in catabolic and metabolic signaling, lysosomes can also function as secretory compartments releasing their luminal content in the extracellular space in a calcium-dependent process (Blott and Griffiths, 2002; Xu et al., 2012; Kimmelman and White, 2017; Buratta et al., 2020). The lysosomal calcium-channel TRPML1 is activated by the metabolic stress conditions typical of cancer cells. Its activation promotes mTORC1 activity and ATP release via lysosomal exocytosis (Liu et al., 2012; Takai et al., 2012; Machado et al., 2015; Naegeli et al., 2017; Xu et al., 2019). Extracellular ATP interacts with purinergic receptors on the plasma membrane, and acts as cancer invasion stimulator by activating Rho GTPase-dependent pathways and upregulating the expression of MMPs (Zhang et al., 2010; Li et al., 2013).

Recent evidence demonstrates that the presence of mitochondria at cell protrusions stimulates ATP-driven actin polymerization to drive cell motility and invasion during *Caenorhabditis elegans* development, even in absence of MMPs (Kelley et al., 2019). This indicates special energy requirement in protruding regions. In line with that, a connection between intracellular mitochondrial trafficking and energy gradients has been described, where ATP:ADP ratio changes depending on positioning and density of mitochondria (Altieri, 2017; Schuler et al., 2017). Long-range mitochondrial trafficking relies on microtubule-associated molecular motors kinesins and dyneins as well as on the mitochondrial Rho-GTPase Miro1. In *Miro1*-deficient mouse embryonic fibroblasts (MEFs), mitochondria reposition to the perinuclear area, which correlates with high ATP production in this region. This inhibits energy-demanding processes such as protrusion formation and focal adhesion dynamics at the cell periphery, resulting in decreased cell migration and invasion (Schuler et al., 2017). Conversely, in migrating and invasive cancer cells, mitochondria accumulate at the leading edge (Arismendi-Morillo et al., 2012). Thus, the traffic and dynamics of mitochondria are coupled to the localized energy demand at the protruding cell front for focal adhesion dynamics, cell membrane dynamics and invasion.

ATP consumption at the leading edge promotes mitochondria trafficking with a positive feedback mechanism that depends on the energy sensor AMPK (Cunniff et al., 2016; Furnish and Caino, 2020). This is in agreement with evidence showing that some key glycolytic enzymes are located at the plasma membrane of invasive cells (Attanasio et al., 2011; Havrylov and Park, 2015; James et al., 2020) where the actomyosin machinery used to displace the ECM relies on the readily available supply of ATP (Oser et al., 2009; van Horssen et al., 2009; Kelley et al., 2019).

Interestingly, tumor exposure to inhibitors of the therapeutic target phosphatidylinositol-3-kinase (PI3K) has shown a unique repositioning of energetically active mitochondria in proximity to focal complexes, which supports membrane dynamics and cytoskeletal remodeling, resulting in increased cell motility and invasion (Caino et al., 2015). Although this response may possibly increase the risk of metastasis, it illustrates the feasibility of targeting mitochondrial reprogramming.

## Concluding Remarks

It is now widely recognized that different metabolic needs are encountered during cancer progression. Therefore, it is of high importance understanding the underlying molecular mechanisms behind this metabolic cancer plasticity for the development of target therapies and also to prevent therapy resistance.

The contribution of intracellular membrane transport to the metabolic rewiring in disease progression is, however, still poorly characterized. Many questions remain unanswered due to the limitations of studying cancer cells in their complex tumor environment. However, studies where nutrient access was restricted by pharmacologically altering membrane trafficking have shown positive results, simultaneously blocking lysosomal degradation of autophagosomes and macropinosomes, and starving cancer cells to death (Kim et al., 2016). In line with this, ongoing studies with agents that target scavenging, macropinocytosis, autophagy or lysosomes seem to be promising (Towers and Thorburn, 2017; Towers et al., 2020). For example, recently developed lysosomal inhibitors for cancer therapy can inhibit multiple lysosomal activities needed for tumor cell survival and growth (Rebecca et al., 2017). Therefore, future research should further explore the molecular mechanisms of intracellular trafficking characterizing tumor initiation, progression and metastasis in relation to the different cellular metabolic needs as these aspects could help to identify new ways or targets for therapy.

Dormant cancer cells are one of the most threatening aspects of cancer and can lead to reoccurrence of metastatic tumors after a long period of latency. Tumor cell dormancy can be induced by nutrient deprivation (Jahanban-Esfahlan et al., 2019) but the mechanism behind the revival of the dormant cells remains mainly elusive. Therefore, further investigation is required to understand whether and how changes in nutrient availability as well as the metabolic adaptation influence this process. Moreover, the role of intracellular trafficking in the re-activation of the dormant cells is still unknown and its characterization may further improve our understanding of tumor dormancy with impact on tumor relapse.

Thus, the tight connection between intracellular trafficking and cell metabolism should be taken into account in the search of novel therapeutic targets for a more integrated cancer therapy.

## Author Contributions

All authors listed have made a substantial, direct and intellectual contribution to the work, and approved it for publication.

## Conflict of Interest

The authors declare that the research was conducted in the absence of any commercial or financial relationships that could be construed as a potential conflict of interest.
